# A Case of Perilymphatic Fistula with Inner Ear Anomaly Diagnosed Preoperatively by the Cochlin-Tomoprotein Detection Test

**DOI:** 10.1155/2020/9476915

**Published:** 2020-03-09

**Authors:** Kana Lee, Naoki Ochi, Kohei Yamahara, Kunihiko Makino, Tetsuo Ikezono

**Affiliations:** ^1^Department of Otolaryngology, Shin-Suma General Hospital, Kobe, Japan; ^2^Department of Otolaryngology and Head and Neck Surgery, Shizuoka City Shizuoka Hospital, Shizuoka, Japan; ^3^Department of Otolaryngology, Saitama Medical University, Saitama, Japan

## Abstract

We present a case of perilymphatic fistula (PLF) with inner ear anomalies having sudden, progressive sensorineural hearing loss and describe the fistula repair surgeries. We focus on the diagnosis methods of PLF and clinical course of PLF with inner ear anomaly. The cochlin-tomoprotein (CTP) detection test is very useful for the surgeons to encourage the earlier operation to sudden hearing loss cases. It is also helpful to define the diagnosis of PLF after operation. We could not get the good result as to hearing from the fistula repair surgery mainly because surgery was held 1 month after the onset. The results of the case, as well as recommendations of other reports, suggest that patients with sudden sensorineural hearing loss and PLF may need repair surgery within at most 2 weeks from the onset. We describe how to diagnose PLF more accurately using CTP detection combined with intraoperative findings.

## 1. Introduction

The diagnosis of perilymphatic fistula (PLF) is controversial. The variability among patients makes PLF difficult to diagnose, not only preoperatively but also intraoperatively. Oozing of the exudate can occur in the middle ear after tympanomeatal flap elevation, leading to a false diagnosis of PLF. In collaboration with ImmunoBiological Laboratories, Gunma, Japan, we developed an enzyme-linked immunosorbent assay kit to detect cochlin-tomoprotein (CTP) using polyclonal antibodies. CTP exists only in the perilymph, and not in the serum, saliva, or cerebrospinal fluid. The CTP detection test is very useful to diagnose PLF and encourages the surgeons to explore the middle ear.

To the best of our knowledge, there have been no cases of PLF with inner ear anomaly diagnosed preoperatively by the CTP detection test. Without the CTP detection test, sensorineural hearing loss with inner ear anomalies is not considered to be linked to PLF. We report the clinical course of a patient with sensorineural hearing loss with inner ear anomaly and PLF.

## 2. Case Reports

The study was performed at Shin-Suma General Hospital in accordance with the institutional ethical standards.

### 2.1. Case

**Figure 1:**
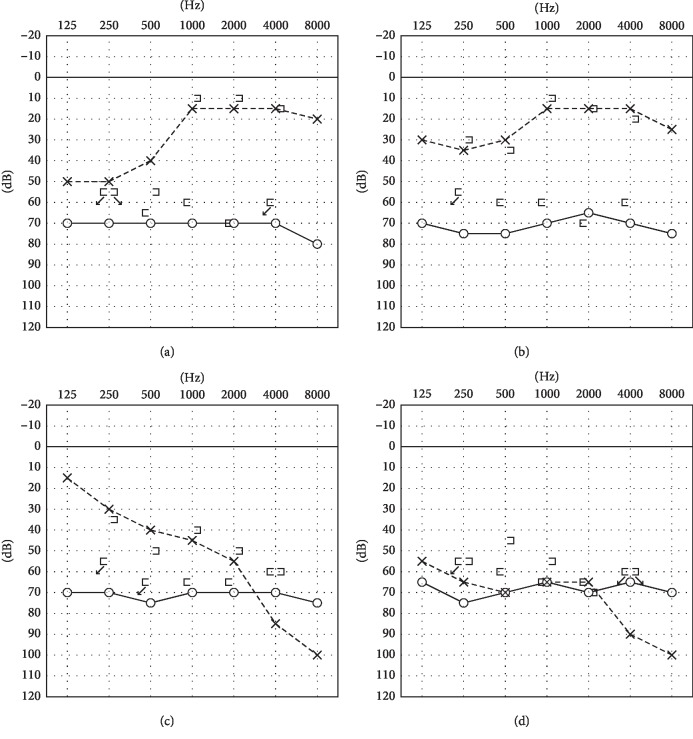
Pure tone audiometry of case 1 before surgery (left ear). (a) Low-tone sensorineural hearing loss (21.3 dB). (b) Hearing had once gotten better (13.8 dB). (c) Exacerbations and remissions (46.3 dB). (d) The hearing suddenly worse (66.3 dB).

**Figure 2:**
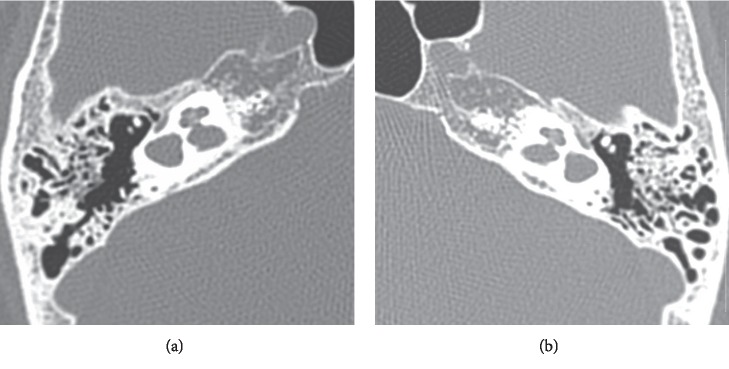
HRCT. The fusion of the vestibules and the lateral semicircular canals. (a) Right ear. (b) Left ear.

**Figure 3:**
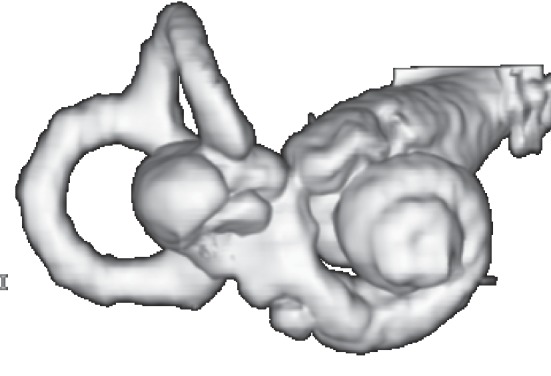
3D fast imaging employing steady-state acquisition-cycled phases (FIESTA-C) magnetic resonance imaging (MRI). The fusion of the vestibules and the lateral semicircular canals.

A 61-year-old woman with blocked ear and low-tone sensorineural hearing loss (21.3 dB) (Figure 1(a)) in the left ear was admitted to the hospital in 2007. She had previously had hearing loss in the right ear (67.5 dB). High-resolution computed tomography (HRCT) showed hypoplasty of the lateral semicircular canals along with fusion of the vestibules and the lateral semicircular canals (Figures 2(a) and 2(b)), but there was no pneumolabyrinth. A 3.0 Tesla magnetic resonance imaging (MRI) and FIESTA-C providing high signal from CSF based on T2/T1 contrast showed no vestibular schwannomas. FIESTA-C used in the 3D mode provided the fusion of the vestibules and the lateral semicircular canals in more detail (Figure 3).

Her hearing had improved of the left (13.8 dB) (Figure 1(b)) after treatment with dexamethasone. There was acute low-tone sensorineural hearing loss of the left with common cold in 2008, and it improved without any medication. There had been gradual sensorineural hearing loss of the left from 2010 to 2013 (approximately 46.3 dB) (Figure 1(c)). She was recommended to undergo exploratory tympanotomy for PLF because she had inner ear anomalies which indicated the presence of PLF. She felt sudden worsening of sensorineural hearing loss of the left with blocked ear and tinnitus like a stream in 2015 and hearing in the left ear declined suddenly (66.3 dB) (Figure 1(d)) to almost the level of the right ear without apparent cause. She had no dizziness or vertigo although she had clockwise nystagmus to the left in the supine position. The fistula test was negative, with no schwannomas on MRI again. The CTP detection test which is used before and within the fistula repair surgery as a biochemical marker for PLF in Japan [1] to confirm the PLF was performed by tympanostomy, while intravenous dexamethasone was administered with the patient in the head-up resting position (using the criterion defined by receiver operating characteristic analysis with the Youden index for CTP, we defined the cutoff criteria as∼0.4 < CTP, negative; 0.4 ≦ CTP < 0.8, intermediate; and 0.8 ≦ CTP, positive [2]). Her hearing showed no improvement. The results of the CTP detection test were positive (1.09), based on tympanostomy before surgery, indicating the presence of PLF. She finally agreed to undergo surgery after the positive result of the CTP test with tympanotomy, though she had refused it for a long time. Exploratory tympanotomy under general anesthesia was performed 1 month after the sudden worsening. Serous liquid continuously leaked and pooled in the middle ear between the stapes and the tympanic portion of the facial nerve during the operation. Spongel and fibrin glue were used to seal the fistula in the oval window and round window after the removal of the fibrous tissue of the round window niche. Hearing loss and positional nystagmus did not improve; however, tinnitus like a stream vanished. CTP detection was positive preoperatively (1.09) and intraoperatively (0.85). We could follow-up only five months after operation because lung cancer was found. There was no improvement of hearing on her last visit. We would recommend the hearing instruments for more severe hearing loss if we could have followed up her.

## 3. Discussion

PLF is defined as an abnormal communication between the perilymphatic space and the outer space in the temporal bone, such as the middle ear or intracranium. Spontaneous PLF without any obvious cause exists although most PLF is caused by external injuries. Middle and inner ear abnormalities are also linked to PLF [3], and patients with multiple meningitis who may have PLF combined with inner ear anomalies have to be treated carefully. Weber et al. reported that middle ear and inner ear malformations were found in up to 86.3% of patients with PLF during surgery. The detection of an inner ear abnormality by CT in a patient with symptoms suggestive of PLF may encourage the surgeon to explore the middle ear [3].

The diagnosis of PLF, both preoperatively and intraoperatively, is clinically very difficult. In some cases, pneumolabyrinth or air bubbles in the inner ear detected by CT may suggest the presence of PLF [4, 5]. The most accepted diagnostic method is intraoperative visualization of perilymphatic leakage in the middle ear, which depends on the subjective judgment of the operator. Some tests, such as beta-2 transferrin and intrathecal fluorescein, have been suggested to confirm PLF [6], but no definitive diagnostic tools are available. Tests for beta-2 transferrin were positive in 66% of patients with PLF [7], while other tests were positive in only 5% [8].

CTP, a protein that is present only in the perilymph, and not in the serum, saliva, or cerebrospinal fluid, was first identified in 2001 [9] and has proven useful for diagnosing PLF. Middle ear lavage with 0.3 ml of saline was performed three times during tympanostomy or tympanotomy. After we cut the tympanic membrane, 0.5 ml syringe with blunt needle filled with 0.3 ml saline is inserted in the middle ear. We collected the saline three times gently and it was centrifuged. The expression of CTP, a short 16-kDa isoform, in the middle ear was analyzed by western blotting using the anti-CTP antibody.

We perform exploratory tympanotomy and PLF repair surgery for the patients with sudden and progressive hearing loss, which is resistant to conservative treatment, or chronic dizziness that is not cured. In this inner ear anomaly case, the patient suffered from sudden sensorineural hearing loss in the left ear. In addition to the left ear, the patient also suffered from severe sensorineural hearing loss of the right ear and positional nystagmus. Although the patient did not complain of dizziness or vertigo, an objective test was conducted to identify the presence of PLF. Based on the patient's sudden hearing loss, abnormal nystagmus, and positive test results for PLF, the patient underwent corrective surgery.

Surgery is performed from the posterior ear or transcanal approach with the patient under general anesthesia. The oval and round windows are visualized to detect any clear fluid, and middle ear lavage is performed three times very gently to detect CTP. In the absence of the CTP detection test, the leak is confirmed by suctioning gently and observing the reaccumulation of the fluid. We cover the leaks by using fascia and Spongel with fibrin glue when we can apparently find the leaks. If we cannot find them, we graft both oval and round window. In the recurrent cases after surgery, fat from the abdomen is used instead of the fascia.

On the postoperative follow-up of this case, hearing improvement was not achieved. One possibility is that the operation was performed too late for the inner and outer hair cells to be repaired. Hearing loss had progressed gradually for 8 years. There may have been a slight leak for 8 years with exacerbations and remissions, and then a large leak resulted in sudden worsening of her hearing. The patient had refused surgery without proof of PLF, such as a CTP detection test, and finally agreed to undergo surgery after the positive result of the CTP test with tympanotomy. The CTP detection test should have been performed earlier because this test is useful in diagnosing PLF preoperatively. Inner ear anomalies tend to have PLF and fistula repair surgery as one of the options of treatment although hearing loss with inner ear anomalies has tended to be thought as untreatable. The leakage from the oval or round windows or from microfissures can cause a change in the pressure difference from the perilymph to endolymph, eventually causing damage to the inner and outer hair cells. We should have performed surgery earlier, following the recommendation of Seo et al. [10] that surgery is indicated within 2 weeks after the onset of hearing loss. Tinnitus like a stream vanished; however, positional nystagmus remained because there was a possibility that the left inner ear injury as to the vascular system remained and PLF still existed in the right ear.

In the surgeries, we grafted both windows with fibrin glue because congenital PLF is most common at the stapes and the round window [11]. The area around the stapes, especially on the anterior part, as well the round window, should be sealed with Spongel and fascia, even when the apparent fistulas are not confirmed. Leakage is likely to occur around the anterior part of the stapes, which frequently moves when the reflex occurs. The amount of perilymph leakage may be directly proportional to the severity of symptoms, such as sensorineural hearing loss, dizziness, and vertigo. PLFs with inner ear anomalies may exhibit severe leakage. A better result may have been achieved by using fat instead of fascia in such a case having more amount of leakage.

The possibility of hearing loss by PLF should be considered among the patients with inner ear anomaly and sudden, fluctuating, and progressive sensorineural hearing loss. Although we have seen only one case of this detected by CTP detection test preoperatively, we could confirm the presence of PLF preoperatively. In inner ear anomalies with various types of sensorineural hearing loss and vertigo CTP detection test should be performed considering the possibility of PLF. We suspect that earlier inner ear injury with multiple slight leakages leads to inner ear injury that is at first reversible and then nonreversible. We suggest that PLF repair surgery should be performed within at most 2 weeks after the onset of hearing loss, especially in patients with anomalies although it is difficult to stop the leakage in cases of multiple fistulas at locations other than the oval and round windows.
